# RGX-019-MMAE inhibits leukemia progression by targeting MER proto-oncogene tyrosine kinase (MERTK) in acute myeloid leukemia

**DOI:** 10.1186/s13046-026-03657-y

**Published:** 2026-03-28

**Authors:** Anudishi Tyagi, Maryam Siddiqui, Amanda Eckstrom, Isabel Kurth, Shugaku Takeda, Priyanka Sharma, Gautam Borthakur, Bin Yuan, Hussein A. Abbas, Vivek Anand, Jenny Borgman, Steven Kornblau, Abhishek Maiti, V. Lokesh Battula

**Affiliations:** 1 Department of Leukemia, The University of Texas MD Anderson Cancer Center, Houston, Texas USA; 2 Inspirna, Inc., New York, New York USA; 3 Department of Internal Medicine, Massey Comprehensive Cancer Center, Virginia Commonwealth University, Richmond, Virginia 23298 USA

**Keywords:** Myeloid epithelial tyrosine kinase (MERTK), Antibody drug conjugate (ADC), Monomethyl auristatin e (MMAE), Reverse phase protein arrays (RPPA), Acute myeloid leukemia (AML)

## Abstract

**Background:**

Myeloid epithelial reproductive tyrosine kinase (MERTK) receptor is overexpressed in cancers and is associated with poor prognosis. RGX-019-MMAE, a novel humanized IgG1-MMAE antibody-drug conjugate (ADC) (Inspirna, Inc), selectively binds to MERTK with high affinity, resulting in internalization and degradation of the receptor. It then induces cytotoxicity through the release of the payload, MMAE (monomethyl auristatin E), which disrupts mitosis.

**Methods:**

MERTK protein expression was analyzed in 818 AML patients using Reverse Phase Protein Arrays (RPPA). Expression was also assessed by flow cytometry in eight AML cell lines and peripheral blood or bone marrow mononuclear cells from five AML patients. Cell lines with the highest MERTK expression were treated with varying doses of RGX-019-MMAE or naked antibody for 120 h, and viability was measured using CellTiter-Glo 2.0. Similarly, primary cells from five AML patients were treated to assess the anti-leukemic effect of RGX-019-MMAE. Further, the combinatorial effects of RGX-019-MMAE with venetoclax (BCL2 inhibitor) were evaluated in vitro.

**Results:**

Reverse-phase protein array in 818 primary AML samples revealed significantly high MERTK protein expression in monocytic acute myeloid leukemia (AML), especially in those with PTPN11, RAS, CEBPA mutations, t (9;11) translocation, and high WBC count, suggesting its potential as a therapeutic target in AML. We also observed varying degrees of MERTK expression in AML cell lines, with highest expression in Kasumi-1 and OCI-AML3. Treatment of these cell lines with the anti-MERTK antibody-drug conjugate RGX-019-MMAE resulted in significantly more leukemic cell killing than the control antibody in a dose-dependent manner. We validated this finding in MERTK-expressing primary AML samples expressing MERTK. Interestingly, RGX-019-MMAE had no effect on normal hematopoietic stem cells’ clonogenic potential. Further, treatment with RGX-019-MMAE inhibited AML progression in vivo and significantly prolonged survival of AML xenograft-bearing mice in a dose-dependent manner. Moreover, treatment with RGX-019-MMAE sensitized AML cells to venetoclax in a dose-dependent manner.

**Conclusion:**

MERTK is overexpressed in AML and could serve as a therapeutic target. Furthermore, RGX-019 MMAE can be used as a novel therapeutic approach for treating AML, especially in treating monocytic subsets of AML.

**Graphical Abstract:**

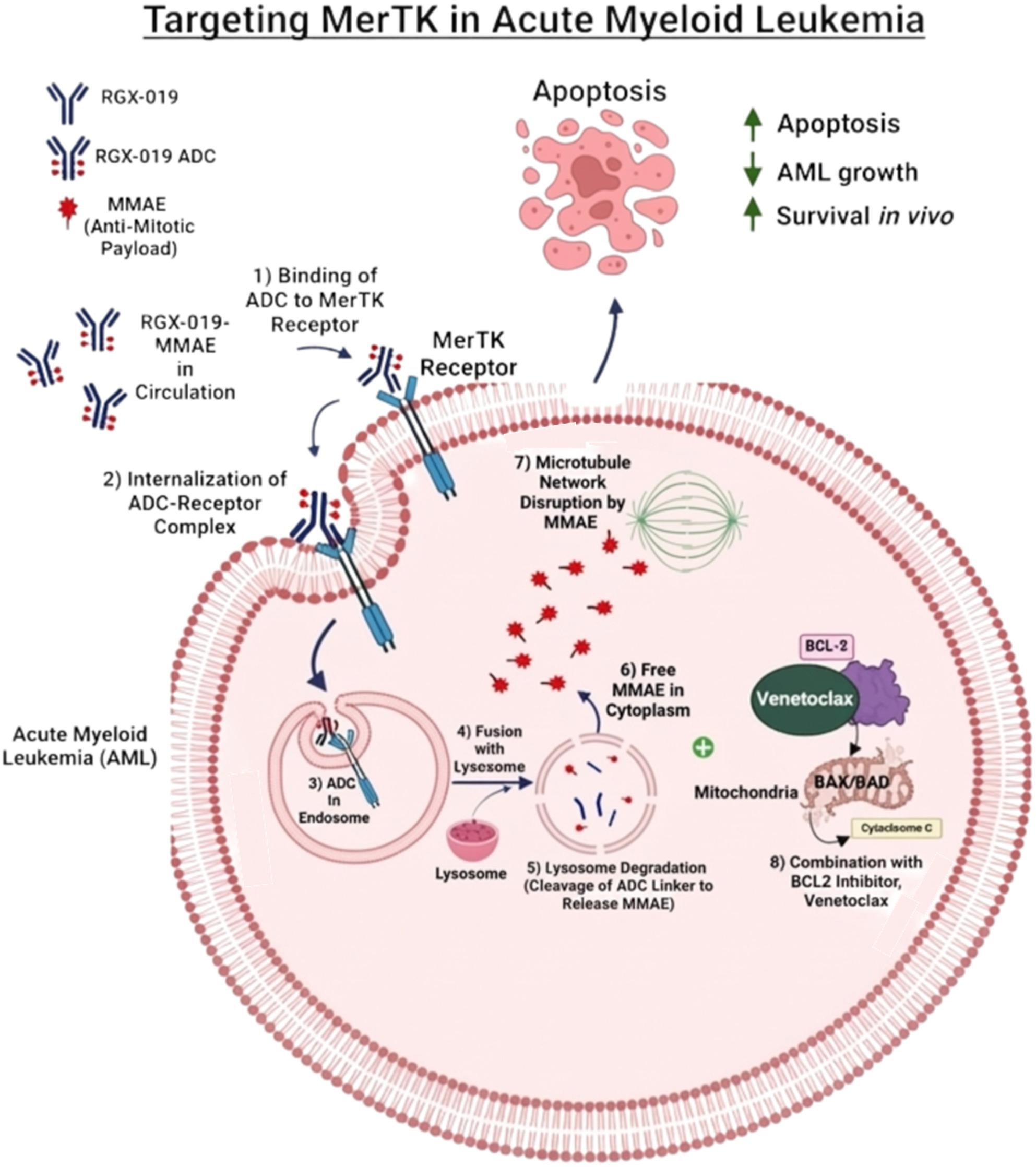

**Supplementary Information:**

The online version contains supplementary material available at 10.1186/s13046-026-03657-y.

## Background

Acute myeloid leukemia (AML), a molecularly heterogenous, aggressive cancer, is characterized by a block in myeloid differentiation that leads to the uncontrolled expansion of myeloblasts in the bone marrow [1]. AML is the most common type of leukemia in adults, and its age-adjusted incidence is approximately 4.3 cases per 100,000 people each year in the United States [2]. Despite recent developments in targeted and combination therapy approaches, some subgroups of AML do not respond or develop resistance over time [3].Therefore, there is a desperate need to develop new therapeutic approaches to target aggressive AML.

One promising approach to target AML is the use of antibody-drug conjugates (ADCs) [4]. An ADC is composed of a monoclonal antibody, which is attached to a cytotoxic payload via a chemical linker [5]. Unlike conventional chemotherapy, ADCs are more specific in reaching the therapeutic target as they release the cytotoxic payloads directly into the target-expressing cancer cells [6]. Indeed, ADCs with targeted chemotherapeutics have shown greater effectiveness than chemotherapy regimens alone [7, 8].

MERTK (MER proto-oncogene tyrosine kinase) belongs to the TAM (Tyro3, Axl, MERTK) family of kinase receptors [9]. MERTK’s binding to growth arrest specific 6 (GAS6) and protein S ligands induces MERTK autophosphorylation and activation of downstream signaling, which includes phosphorylation of AKT and ERK [10]. MERTK plays a crucial role in progression of cancer due to its role in cancer cell survival signaling [11]. In most AML samples (80%) from adult and pediatric patients, MERTK is overexpressed compared with its expression in healthy bone marrow precursor cells [12–14]. It has been reported that targeting MERTK by shRNA or small molecule inhibitors led to tumor cell apoptosis and prolonged survival in mouse models [12]. Several studies reported its important role in mediating resistance to targeted therapies [15–18]. In ALL models, activation of the MERTK signaling pathway promoted chemoresistance [19], and shRNA-mediated MERTK knockdown increased sensitivity to cytotoxic chemotherapy agents [13, 20]. Given its significant role in cancer, targeting MERTK has emerged as a potential therapeutic approach. Although MERTK-targeted monoclonal antibodies have shown promising activity in solid cancers [11, 21], the effectiveness of targeting MERTK using ADCs in AML is not known.

Therefore, we hypothesized that targeting MERTK using a novel anti-MERTK ADC, RGX-019-MMAE, would be effective in killing leukemic cells and inhibiting leukemia progression. We measured MERTK expression in AML cell lines and AML patient samples and confirmed its association with poor prognosis. Further, we investigated the cytotoxic effects of RGX-019-MMAE in AML in vitro and in vivo. In addition, we investigated the effect of RGX-019-MMAE on normal hematopoietic stem cells. Finally, we investigated the effect of RGX-019-MMAE in combination with approved targeted chemotherapy in AML cells.

## Methods

MERTK expression in AML cell lines.

### Cell culture

Kasumi-1, THP-1, MV4-11, and U937 AML cells were purchased from ATCC (Manassas, VA), and MOLM-13, MOLM-14, OCI-AML3, and OCI-AML2 cells were obtained from DSMZ (Braunschweig, Germany). These cell lines were cultured in RPMI1640 medium (Corning, Manassas, VA, 15-040-CV) supplemented with 10% fetal bovine serum (Gibco, Waltham, MA, 26140-079), 1% penicillin/streptomycin (Sigma-Aldrich, St. Louis, MO, P4333), and 1% L-glutamine (Corning, 25-005-CI). The medium was changed twice weekly. Tests for Mycoplasma contamination of leukemic cells are performed in our laboratory every 4 to 6 months. To confirm purity, all cell lines are fingerprinted every 6 months at the MD Anderson’s Characterized Cell Line Core.

### Flow cytometry

We performed flow cytometry to measure MERTK protein expression in AML cell lines and AML patient samples. Briefly, 1 × 10^6^ cells were washed twice with flow cytometry buffer (phosphate-buffered saline plus 2% fetal bovine serum), incubated in the dark with anti–MERTK-APC (BioLegend, San Diego, CA) antibody for 30 min on ice, washed in flow buffer, and counterstained with 4′,6- diamidino-2-phenylindole (DAPI; 0.5 µg/mL; Thermo Fisher Scientific, Waltham, MA) to exclude any dead cells. The data were acquired on MACS Quant16 (Miltenyi Biotech, Bergisch Gladbach, Germany) and Gallios (Beckman Coulter, Brea, CA) flow cytometers. For each sample, a minimum of 10,000 events were acquired, and data were analyzed using FlowJo software v10 (FlowJo, LLC).

### MERTK expression in AML patient samples

#### Patient sample characteristics

Primary AML cells were derived from peripheral blood samples collected from 818 patients between January 2012 and August 2023. The sample collection was conducted according to a protocol approved by the Institutional Review Board at The University of Texas MD Anderson Cancer Center (Protocol PA18-0129), and all study participants provided written informed consent. Patient characteristics were extracted from MD Anderson Cancer Center’s electronic health record system (EPIC, Epic Systems Corporation). White blood cell (WBC) counts and blast percentages for each patient were recorded by the MD Anderson Leukemia Sample Bank.

#### Reverse-phase protein array

Reverse-phase protein array (RPPA) was performed to measure MERTK expression in 818 AML patients’ samples. RPPA was performed in the MD Anderson RPPA Core Facility as described previously [22–24]. Briefly, whole-cell lysates were subjected to five serial 2 × dilutions (1:1, 1:2, 1:4, 1:8, and 1:16) and printed onto nitrocellulose-coated glass slides. To determine MERTK protein expression levels, slides were probed with anti-MERTK monoclonal antibody. The primary antibody, together with secondary antibodies conjugated to an infrared molecule, were validated as previously described [25]. Stained slides were quantitated with Microvigene (Version 3.4, VigeneTech, Carlisle, MA), and expression was normalized to normal bone marrow derived CD34 + cells. More specifically, the mean expression of normal bone marrow was normalized to zero, and the values of each AML sample were expressed in Log_2_-fold-change values compared to normal bone marrow.

### Effect of RGX-019-MMAE on AML cell lines and primary AML cells

#### RGX-019 synthesis and generation of RGX-019-MMAE

Mouse IgG1/kappa light chain MERTK-specific monoclonal antibodies were generated in mice by immunization with Fc-tagged human MERTK peptide (R&D Systems, Minneapolis, MN) USA). Hybridomas were generated from mice that showed MERTK-specific antibodies in serum and were further tested based on a triple screen that included ELISA assays to measure binding to MERTK, followed by ability to block Gas6 binding to MERTK and finally a counter screen for Axl binding. Antibodies from top candidate clones with single digit nanomolar affinity to MERTK were scaled up in the Mab Express Bioreactor and further tested in endothelial recruitment assays and in vivo anti-tumor efficacy. Antibodies were generated by antibody solutions.

The clone with highest specificity and affinity was then selected for humanization by WuXi Biologics (Shanghai, China). Humanization was conducted by fusing the humanized VH domain to human IgG1 segments in the heavy chain and fusing of the humanized VL domain to human Ig kappa CK domain in the light chain. The constructs were further codon optimized for recombinant expression in CHO cells. Scale up synthesis of RGX-019 was conducted using WuXian Express (WuXi). RGX-019 was purified from filtered cell lysates by chromatography on a MabSelect SuRe (50/20) column, followed by a Superdex200 (50/88) column. Purity was confirmed by SDS-PAGE (> 95%) and SEC-HPLC (100%) analysis. Endotoxin levels were < 1 EU/mg.

RGX-019 was conjugated with a linker-payload consisting of the microtubule inhibitor MMAE attached to a protease-cleavable linker containing a PEG spacer group. The antibody was first partially reduced with TCEP, followed by site-specific cysteine conjugation to the linker–payload, at an average drug-to-antibody ratio (DAR) of 4:1. DAR, purity, and molecular identity of the ADC were confirmed by hydrophobic-interaction chromatography (HIC), size-exclusion chromatography (SEC), and liquid chromatography–mass spectrometry (LC-MS) analysis, respectively. Antibody drug conjugation was conducted by Abzena (Cambridge, CB22 3AT, United Kingdom).

#### RGX-019 internalization assay (Nomo-1, Kasumi-1)

RGX-019-MMAE was labeled with pHrodo Red, a pH-sensitive dye, using pHrodo Red Microscale Labeling Kit (ThermoFisher, P35363) according to the manufacturer’s instructions. Internalization of the antibody was determined by flow cytometry detecting pHrodo fluorescence, which is minimal at neutral pH and maximal in acidic environments, such as the lysosomes. Hundred thousand Nomo-1 (p4) or Kasumi-1 (p5), cells were plated in 400 µL cell culture medium in 24-well plates. The next day, 20 µL of Human TruStain FcX Fc Receptor blocking solution (Biolegend) at 1:20 dilution was added and mixed well. After 10 min. incubation, 400 µL medium with 2 × concentration (20 nM) of RGX-019-MMAE was added and cells incubated for 6 h. The cells were collected, washed twice, and resuspended in 100 µL FACS buffer (2% BSA, 10 mM EDTA, 25 mM HEPES, 0.1% sodium azide in Ca/Mg-free PBS). The pHrodo signal was quantified with flow cytometer (BD LSR Fortessa) and analyzed with FlowJo, and then normalized by Degree of Labeling (DOL).

#### Cell viability assay

Kasumi-1, MV4-11, MOLM-13, MOLM-14, and OCI-AML3 AML cell lines and AML primary cells were seeded at 5 × 10^4^ per well in 96-well plates. A 100 µL aliquot of cell suspension was dispensed into each well of a 96-well culture plate, followed by the addition of varying concentrations of MMAE (0.5, 1, 2 nM), RGX-019 and RGX-019-MMAE (0, 3.75, 7.5, 15, 30, 60, and 120 nM) to achieve a final volume of 150 µL. Each concentration was tested in triplicate. The 96-well plates were then enclosed and incubated for 5 days at 37 °C in a 5% CO_2_ incubator. Following this incubation period, 100 µL of CellTiter-Glo 2.0 (Promega, Madison, WI) was added to each well, and the culture plates were further incubated for 15 min, shielded from light. The intracellular ATP levels were quantified in cells using a luciferase-based assay per the reagent’s manufacturer instructions. The luminescent intensity was measured using an Infinite M200 reader (TECAN, Männedorf, Zürich, Switzerland).

#### Primary NK and T cell culture

Buffy coats from healthy individuals were purchased from the Gulf Coast Regional Blood Center (Houston, TX). PBMCs were isolated using LymphoSep density separation (Corning, Manassas, VA). Natural Killer (NK) and T cells were generated and expanded from PBMCs as previously described [26]. Briefly, for natural killer cells, first CD3⁺ T cells were depleted through negative magnetic selection using CD3 beads (Miltenyi Biotec, Auburn, CA). The resulting cell fraction was cocultured with irradiated K562 feeder cells in the presence of IL-15 and IL-21. T cells were activated using anti-CD3 and expanded into IL-2 containing medium. Both activated NK and T cells were either treated with RGX-019-MMAE or unconjugated RGX-019 with a concentration range of 12.5 to 200 nM for in vitro cytotoxicity assays.

#### Colony formation assay

The colony formation assay was performed as described previously [27]. Briefly, healthy donor buffy coats were purchased from the Gulf Coast Regional Blood Center (Houston, TX). Peripheral blood mononuclear cells were isolated using Lymphocyte Separation Medium (Corning). Isolated mononuclear cells were counted, followed by preparation of the suspension of 1 × 10^5^ cells in 400 µL of complete RPMI-1640 (Sigma-Aldrich), supplemented with 10% fetal bovine serum in 15-mL Falcon tubes. Then, a total of 4 mL of MethoCult H4435 Enriched Methylcellulose Medium (Stem Cell Technologies, Vancouver, Canada, 04435) was transferred to each Falcon tube using a syringe with blunt-end 16-gauge needle (Stem Cell Technologies, Vancouver, Canada, 28110) and the cells and methylcellulose medium were vortexed for 1 min to create a uniform suspension. Different concentrations of RGX-019 antibody or RGX-019-MMAE (i.e., 25, 50, 100 and 200nM) were added to Falcon tubes followed by vortexing to ensure all components are mixed uniformly and thoroughly. After 15 min of rest, 3 mL of methylcellulose was distributed between 3 bottom-gridded culture plates (Stem Cell Technologies, Vancouver, Canada 27370) with an aimed density of 2.5 × 10^4^ cells/mL of methylcellulose/well using a 3-mL syringe with 18-gauge 1½ inch needle. Cells were allowed to grow for 14 days before colonies were manually counted.

#### Flow cytometric analysis of combination treatments

To assess cytotoxicity by RGX-019-MMAE in combination with approved AML targeted therapy, OCI-AML3 and THP-1 cell lines were treated with 200 nM concentrations of venetoclax in combination with 100 nM and 200 nM of RGX-019-MMAE or RGX-019 for 5 days in 96-well flat-bottom plates (Falcon, 353072). Each sample was then transferred to a round-bottom plate (Corning, 3799) for staining with annexin V-FITC (BioLegend, 640945), and 0.25 µg/mL DAPI (Invitrogen, 1890543) in annexin V binding buffer (BioLegend, 422201). Flow cytometric analysis was performed using a MACS Quant16 flow cytometer.

#### In vivo mouse studies

Male 6- to 8-week-old NSG mice (NOD.Cg-Prkdc^*scid*^Il2rg^*tm1Wjl*^/SzJ,, *n* = 35) were purchased from The Jackson Laboratory (Bar Harbor, ME). All mice were housed in the Animal Core Facility at MD Anderson Cancer Center. All mouse experiments were performed in accordance with MD Anderson Institutional Animal Care and Use Committee guidelines and were approved by the committee. For the mouse xenograft models, we generated two models, one using MOLM-13 AML cells and one with MOLM-14 AML cells. Both MOLM-13 and MOLM-14 AML cells were transduced with a retroviral vector encoding the enhanced green fluorescent protein firefly luciferase (eGFP-FFluc) gene [28]. A single-cell clone was first selected based on high eGFP expression and in vitro FFluc activity. eGFP-FFluc–MOLM-13 cells (0.5 × 10^6^ ) were then injected into the lateral tail veins of 6- to 8-week-old male NSG mice. Leukemia engraftment was monitored via bioluminescence imaging (BLI) using an AMI Spectral Instruments Imaging in vivo system (PerkinElmer, Waltham, MA). After engraftment, mice were randomized into seven groups: (1) vehicle (phosphate-buffered saline); (2) RGX-019, 1 mg/kg; (3) RGX-019, 5 mg/kg; (4) RGX-019, 10 mg/kg; (5) RGX-019-MMAE, 1 mg/kg; (6) RGX-019-MMAE, 5 mg/kg; and (7) RGX-019-MMAE, 10 mg/kg. RGX-019 and RGX-019-MMAE antibody were dosed once a week by intraperitoneal injection for 4 weeks. For the MOLM-14 AML xenograft model, eGFP-FFluc–MOLM-14 cells (0.5 × 10^6^ ) were injected into the lateral tail veins of 6- to 8-week-old male NSG mice. Leukemia engraftment was also monitored via bioluminescence imaging (BLI) using an AMI Spectral Instruments Imaging in vivo system (PerkinElmer, Waltham, MA). After engraftment, mice were randomized into five groups: (1) vehicle (phosphate-buffered saline); (2) RGX-019, 1 mg/kg; (3) RGX-019, 5 mg/kg; (4) RGX-019-MMAE, 1 mg/kg; and (5) RGX-019-MMAE, 5 mg/kg. RGX-019 and RGX-019-MMAE antibody were dosed once a week by intraperitoneal injection for 4 weeks. All mice in each model were monitored and euthanized according to protocols approved by the MD Anderson Institutional Animal Care and Use Committee.

### Statistical analysis

The data from live cell imaging apoptosis assays and flow cytometry assays were reported as mean ± standard error. AML cell viability was compared between treatment groups by one-way Welch ANOVA with multiple comparisons and Student unpaired *t*-test. The Mann-Whitney test was used to compare differences between mutated and non-mutated groups, and the Kruskal-Walli’s test was used to compare more than one group in AML cohorts. The log rank test was used to compare Kaplan-Meier survival curves. All figures and analyses were generated using GraphPad Prism 10, except for flow cytometry dot plots, which were generated using FlowJo v10.10.0 software. *p* values lower than 0.05 were considered significant.

## Results

### High MERTK expression in AML and its association with monocytic subtype

**Fig. 1:**
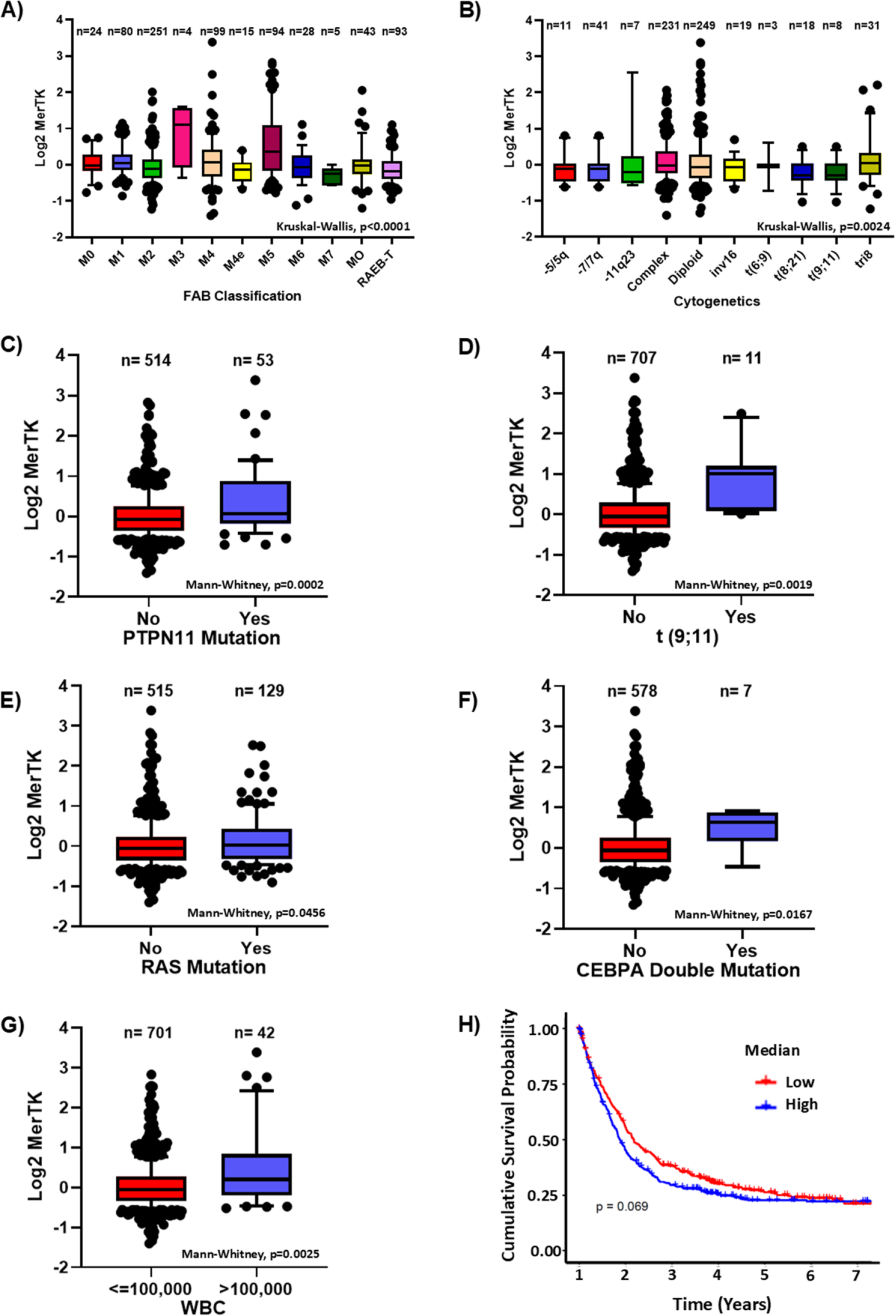
High MERTK expression and its association with the monocytic subtype of AML. **A** MERTK expression in different AML subtypes identified based on the French-American-British classification system (Kruskal-Wallis test). **B** MERTK expression in AML patients with different cytogenetic characteristics (Kruskal-Wallis test). **C**-**F** MERTK expression in AML patients with PTPN11 mutation, t(9;11), RAS mutation, and CEBPA mutation (Mann-Whitney test). **G** Association of MERTK expression in AML patients with low and high white blood cell counts (WBCs, Mann-Whitney test). **H** Overall survival of AML patients divided into high and low MERTK expression (log-rank test)

We analyzed MERTK expression in peripheral blood and bone marrow mononuclear cells by RPPA from patients with AML recruited at MD Anderson Cancer Center. These patients’ baseline characteristics, molecular mutations and translocations are summarized in Table S1 and S2. MERTK expression was high in the total AML cell population compared to normal CD34^+^ cells (Fig. S1). Furthermore, we found that its expression was high in patients with the M3, M4, and M5 subtypes of AML (Fig. 1A) and those with complex karyotype, diploid, inversion 16 and trisomy 8 cytogenetic abnormalities (Fig. 1B). A significant increase in MERTK expression was found in patients with PTPN11 mutations (*p* = 0.0002), t(9;11) translocations (*p* = 0.0019), RAS mutations (*p* = 0.0456), and CEBPA mutations (*p* = 0.0167) compared to the patients who do not carry these mutations (Fig. 1C-F). Additionally, we stratified the patients based on their white blood count (WBC) to explore the prognostic significance of MERTK expression in AML. MERTK expression was higher in patients who had high WBC counts compared to patients with low WBC counts (Fig. 1G). In addition, to examine the clinical significance of MERTK expression in AML, we stratified the patients based on high and low MERTK expression. We observed a trend showing that high MERTK protein expression is associated with inferior overall survival compared to the patients with low MERTK expression (*p* = 0.069) (Fig. 1H). These findings indicate that MERTK is upregulated in the monocytic subtype of AML and that its expression is showing a trend towards the association with poorer prognosis.

### RGX-019-MMAE design and internalization

The development of RGX-019-MMAE has been described previously. The development of RGX-019-MMAE has been described previously [29], demonstrating RGX-019-MMAE’s anti-tumoral activity in a broad range of MERTK-expressing solid tumors through mechanisms involving direct cancer cell killing and stimulation of an anti-tumoral immune response. Briefly, RGX-019, an anti-MERTK humanized monoclonal antibody, is linked to an anti-mitotic payload, monomethyl auristatin E (MMAE). RGX-019-MMAE binds to a unique epitope on MERTK on AML cells and upon binding, induces MERTK receptor internalization and uptake of the antibody-ADC. Once the RGX-019-MMAE is internalized through endosomes and traffics to the lysosome, MMAE is released by cleavage of the linker. To show MERTK-dependent antibody internalization, we treated two AML cell lines, NOMO-1 and Kasumi-1 with low pH-dependent fluorophore pHrodo-labeled RGX-019-MMAE. We also added an Fc block to prevent non-specific uptake of antibodies. The data revealed a robust increase in pHrodo signal in the RGX-019-MMAE treated cells, demonstrating MERTK specific uptake and internalization of the antibody ADC in AML cell lines (Fig S3A).

### RGX-019-MMAE induced cytotoxic activity in MERTK-expressing AML cell lines

**Fig. 2:**
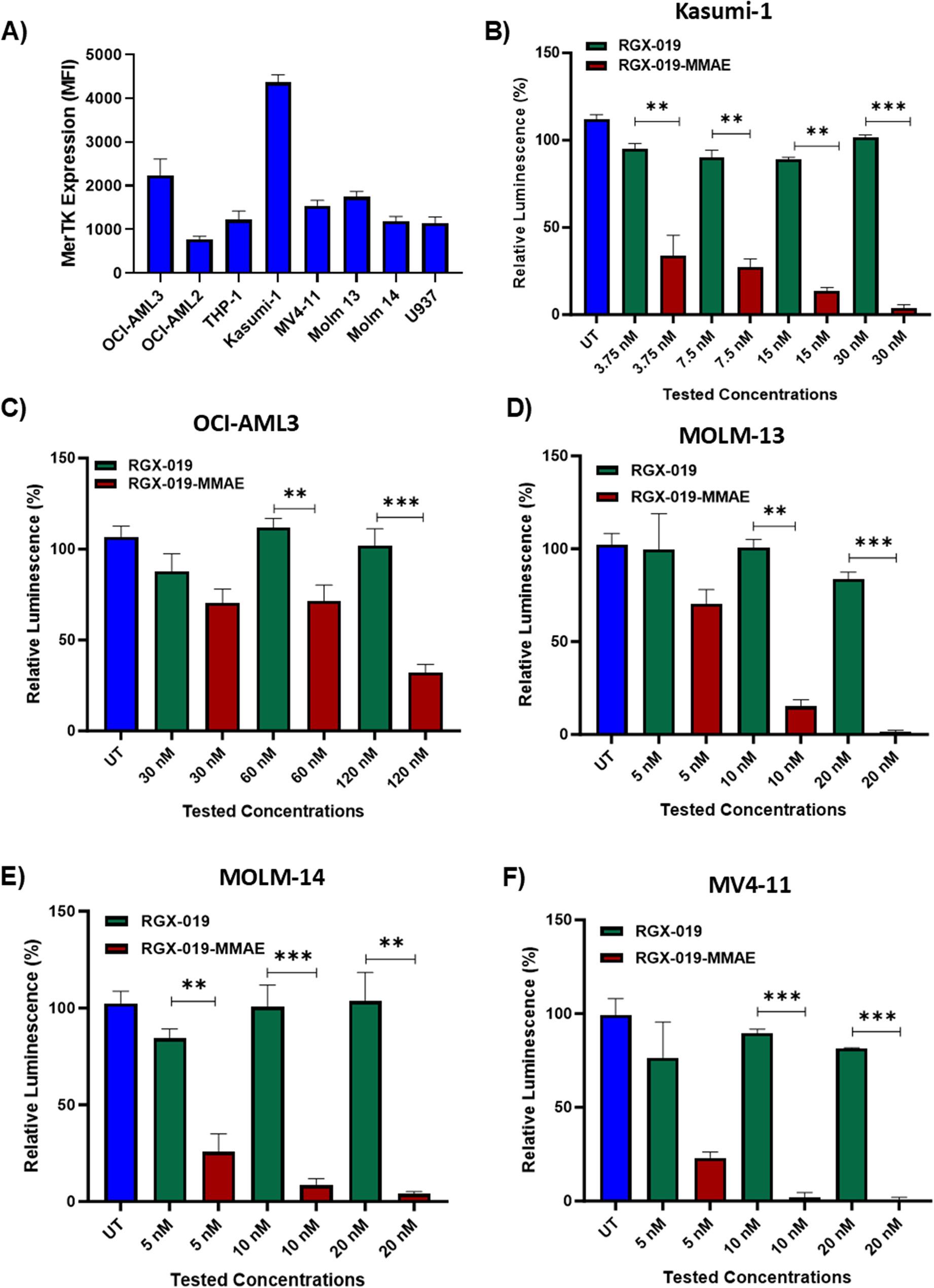
The antibody-drug conjugate RGX-019-MMAE induced cytotoxic activity in MERTK-expressing AML cell lines. **A** MERTK expression in the indicated AML cell lines; leukemic cells (1 × 10^6^) were stained with anti-MERTK-APC antibody, and the expression was measured by flow cytometry. Data are plotted as mean values with error bars representing standard error. **B**-**F** Bar graph showing the percentage of relative luminescence in Kasumi 1 (**B**), OCI-AML3 (**C**), MOLM-13 (**D**), MOLM-14 (**E**), and MV4-11 (**F**) cells treated with the indicated concentrations of RGX-019-MMAE and monoclonal antibody RGX-019. Data are plotted as mean values with error bars representing standard error (Student unpaired *t*-test). *p≤0.05, **p≤0.01, ***p≤0.001, ****p≤0.0001. UT = untreated

To determine the sensitivity of free MMAE payload in AML cells, we treated Kasumi-1 and OCI-AML3 cells with different concentrations of MMAE. We observed ~ 80% cell death in both Kasumi-1 and OCI-AML3, even at 1 nM concentration, suggesting that the AML cell lines are highly sensitive to free MMAE (Fig. S2A-B). We then measured MERTK expression in eight AML cell lines by flow cytometry. We observed varying degrees of MERTK protein expression in these cell lines, with Kasumi-1 and OCI-AML3 expressing the highest levels of MERTK protein (Fig. 2A). We thus treated Kasumi-1 and OCI-AML3 with various concentrations of RGX-019-MMAE or the naked antibody, RGX-019, and observed that RGX-019-MMAE significantly induced cell killing in these cell lines in a dose-dependent manner, compared to the naked antibody (*p* < 0.01, Fig. 2B-C). FLT3 is frequently mutated in AML and its presence is often associated with poor prognosis [30]. We therefore tested FLT3-mutated AML cell lines, including MOLM-13, MOLM-14, and MV4-11, and observed increased sensitivity to RGX-019-MMAE (Fig. 2D-F) with more killing of the cells at concentrations > 20 nM. These findings suggest that RGX-019-MMAE effectively inhibits the expansion of MERTK-expressing AML cells, including those with FLT3 mutations, which remain challenging to treat.

### RGX-019-MMAE induced cytotoxicity in primary AML cells

**Fig. 3:**
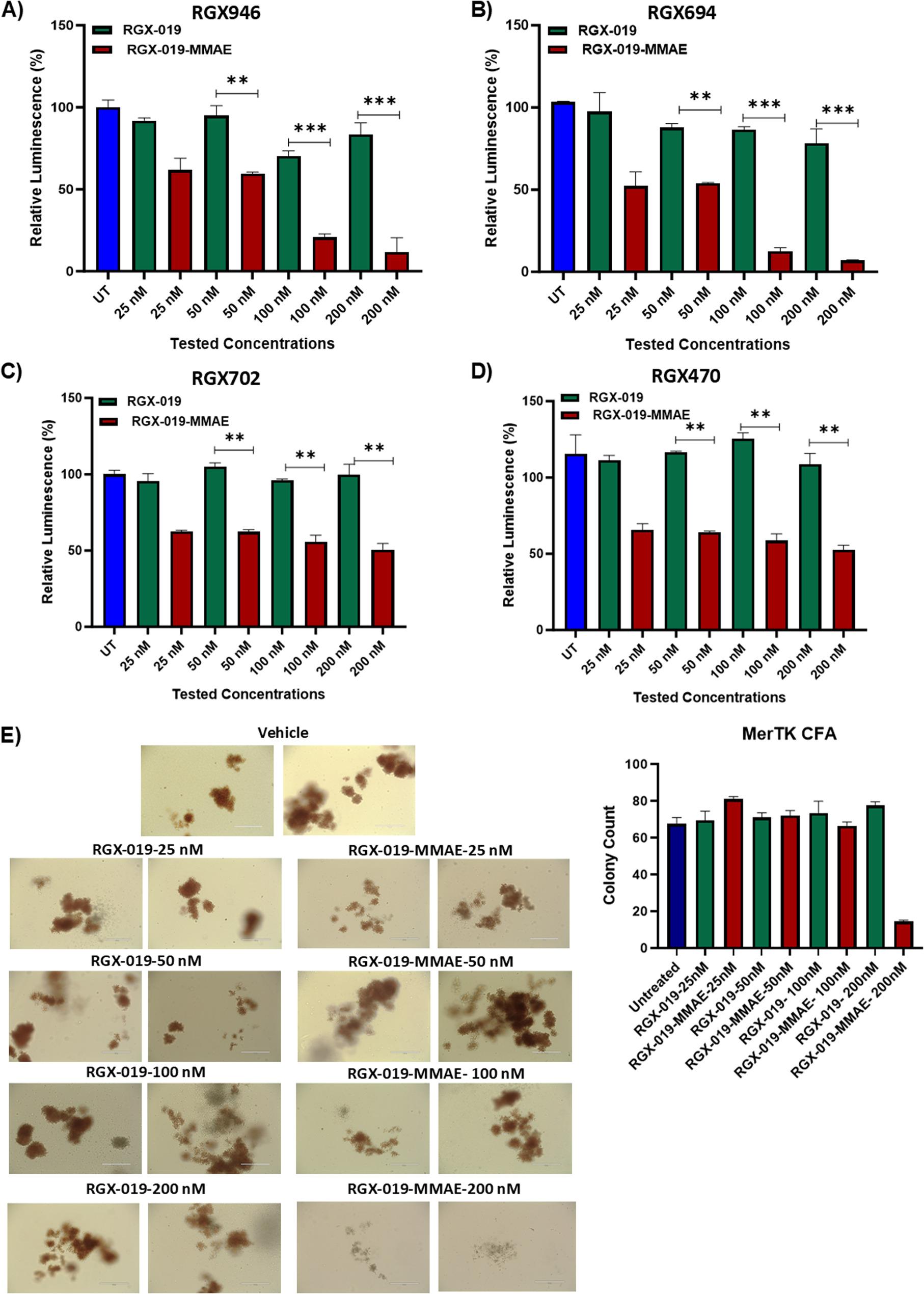
The antibody-drug conjugate RGX-019-MMAE induced cytotoxic activity in MERTK-expressing AML primary cells. **A**-**D** Bar graphs show the percentage of relative luminescence in primary AML cells, RGX946 (**A**), RGX694 (**B**), RGX702 (**C**), and RGX470 (**D**) treated with the indicated concentrations of RGX-019-MMAE or the monoclonal antibody RGX-019. Data are plotted as mean values with error bars representing standard error (Student unpaired *t*-test). **E** Representative figures of colony-forming units for normal peripheral blood mononuclear cells in response to DMSO and indicated concentrations of RGX-019-MMAE and RGX-019. Data are plotted as mean values with error bars representing standard error (Student unpaired *t*-test) *p≤0.05, **p≤0.01, ***p≤0.001, ****p≤0.0001

To validate the cytotoxic effects of RGX-019-MMAE in primary cells, we selected 7 samples from an AML patient cohort of 818 that expressed MERTK for RPPA analysis. The patients’ baseline characteristics are summarized in Table 1 and Table S1, and MERTK expression in selected primary samples is shown in an overlay plot (Fig S4A). We treated MERTK-expressing primary AML cells with varying concentrations of RGX-019-MMAE and the naked antibody, RGX-019. The results showed that RGX-019-MMAE showed significantly increased cell death in a concentration-dependent manner compared with the naked antibody (*p* < 0.01, Fig. 3A-B, S4B). Interestingly, we observed a small subset of primary AML samples that were resistant to RGX-019-MMAE (Fig. 3C-D, S4C-D). To determine the impact of RGX-019-MMAE on normal cells, we evaluated the effect of both the ADC and the naked antibody on the colony-forming ability of progenitor cells using peripheral blood mononuclear cells isolated from a healthy donor buffy coat. We observed no effect on clonogenic potential of healthy cells at concentrations up to 100 nM (Fig. 3E). To evaluate the potential impact of RGX-019-MMAE on immune cells, we performed vitro assays using activated NK cells derived from PBMCs of healthy donors. RGX-019-MMAE treatment did not induce cytotoxicity in NK cells, indicating that these cells are largely unaffected by the ADC. In contrast, treatment with unconjugated RGX-019 at higher doses (100 nM and 200 nM) induced some cytotoxicity (Fig S4E). These findings revealed that RGX-019-MMAE induces concentration-dependent cytotoxic effects in MERTK-expressing primary AML cells, effectively inhibiting their proliferation.

**Table 1:** AML patients characteristics

Sample	Mutations	Mutations	Recent Treatments	Blast%	WBC
RGX946	NPM1	46, XX [20]	status post two cycles of SAHA with idarubicin andara-C chemotherapy	45%	60.2
RGX782	FLT3	46, XX	N/A	45%	60.4
RGX228	N/A	N/A	N/A	N/A	318.2
RGX470	TP53, PTPN11, TET2	46, XX [20]	cytarabine iv 100u/m2/day (200mg) continuouslyfor 7 days, 60 mg/m2 (121 mg) of daunorubicin	68%	75.5
RGX702	FLT3-ITD, PTPN11, RUNX1, TET2, TP53, ASXL1	46, XX [20]	venetoclax tablet 100 mg, oral 16 of 16 cycles,decitabine 37 mg iv 16 of 16 cycles, quizartinib 30mg	47%	31.7
RGX574	JAK2, KIT, KRAS, TP53, DNMT3A, PTPN11	N/A	azacytdine, twice daily fludarabine and ara-c	34%	25.1
RGX694	FLT3-ITD, DNMT3A, TET2, RUNX1, WT1	N/A	Cladribine 4mg/m2, cytarabine 20 mg, sorafenib200 mg	20%	6.6

### Anti-leukemic activity of RGX-019-MMAE in FLT3-mutated xenograft models

**Fig. 4:**
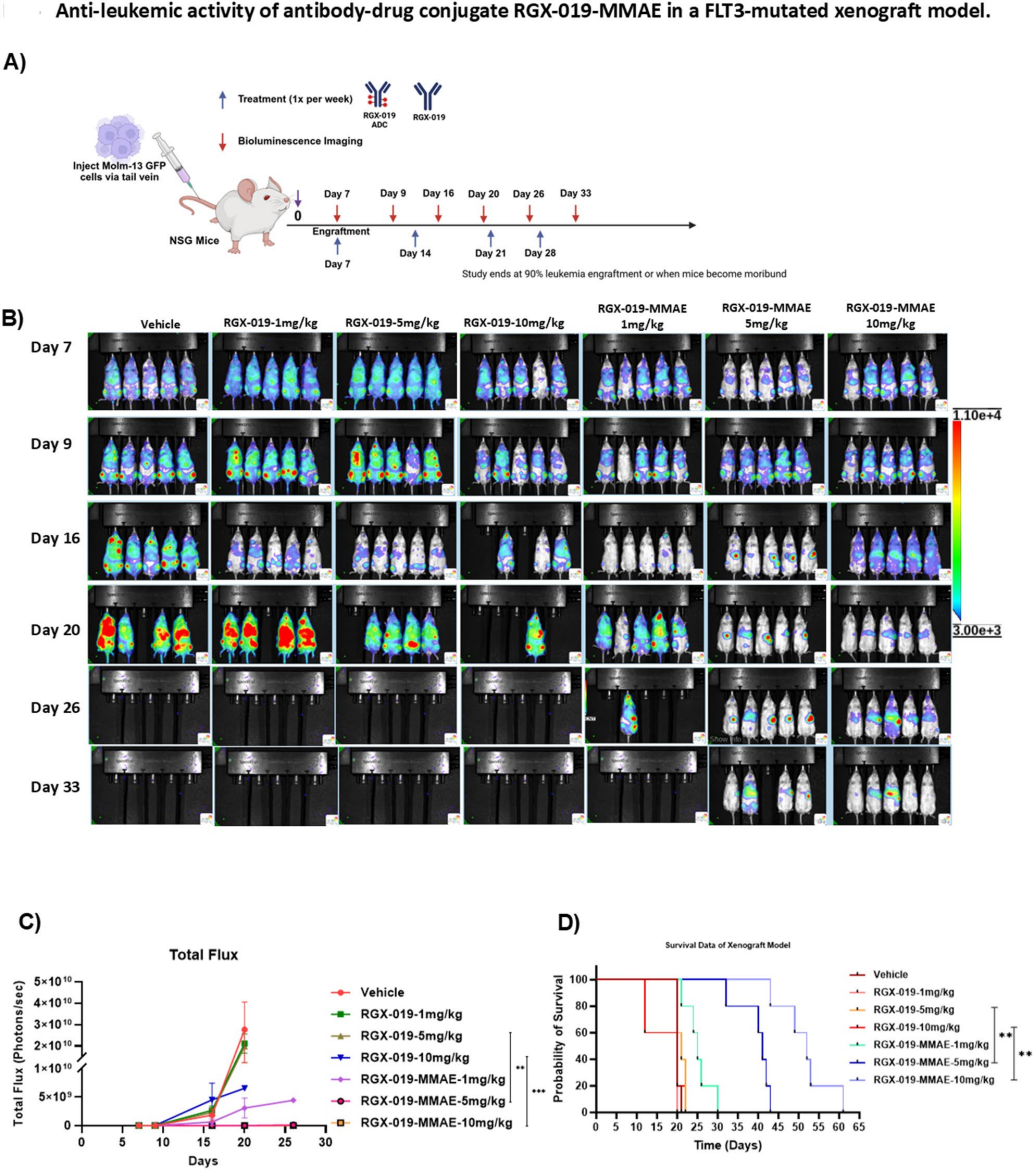
Anti-leukemic activity of antibody-drug conjugate RGX-019-MMAE in a FLT3-mutated xenograft model. **A**) Experimental design: 0.5 million firefly luciferase GFP+ MOLM-13 cells were injected in NSG mice via tail vein, and leukemia engraftment was measured weekly by bioluminescence imaging (BLI). The mice were treated with monoclonal antibody RGX-019 or RGX-019-MMAE at 1, 5, or 10 mg/kg once a week via intraperitoneal injection. **B**) BLI images of the mice treated with indicated doses of RGX-019 or RGX-019-MMAE. **C**) Line diagram showing the BLI total flux (in photons s−1) at day 7, 9, 16, 20, 26, and 33, comparing leukemia progresssion in mice treated with the indicated doses of RGX-019 or RGX-019-MMAE. **D**) Kaplan-Meier survival plot demonstrating overall survival rates in mice treated with RGX-019 or RGX-019-MMAE (log-rank test) *p≤0.05, **p≤0.01, ***p≤0.001, ****p≤0.0001

To evaluate the effect of RGX-019-MMAE on leukemic progression in vivo, we injected MOLM-13 cells expressing firefly luciferase (0.5 million cells per mouse; *n* = 5) in NSG mice via tail vein injection. Seven days later, leukemia disease burden was measured using bioluminescence imaging (BLI). When the leukemia burden reached 1.5 × 10^9^ photons per second, the mice were randomly distributed into seven groups as shown in Fig. 4A. Leukemia disease burden was tracked via BLI every week. Treatment with RGX-019 did not inhibit leukemia progression compared to vehicle treatment. In stark contrast, disease burden was significantly lower in the mice treated with RGX-019-MMAE at both 1 mg/kg and 5 mg/kg doses compared to the RGX-019-treated groups (Fig. 4B-C). Kaplan-Meier survival analysis revealed that mice treated with RGX-019-MMAE had a median survival of 41 days in the 5 mg/kg group and 52 days in the 10 m/kg. In comparison, mice treated with RGX-019 antibody had a median survival of 20 days in the 5 mg/kg group and 25 days in the 10 mg/kg group (*p* < 0.001, Fig. 4D) with extended survival up to 3-fold. Adding on, mice in the vehicle treatment group had a median survival of 20 days. These findings demonstrated that RGX-019-MMAE inhibits leukemia progression in vivo and extends the survival of mice by killing MERTK-expressing cells.

**Fig. 5:**
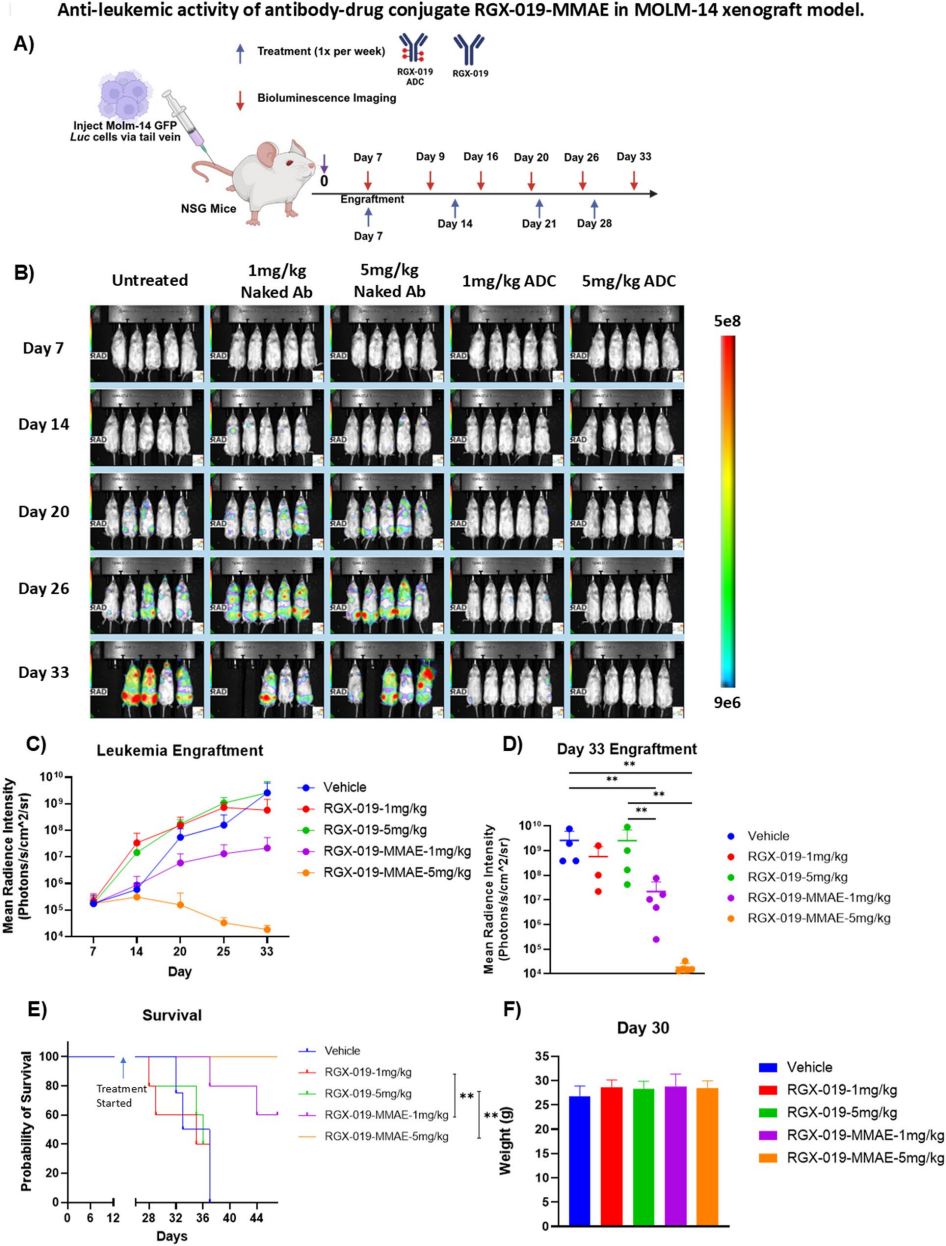
Anti-leukemic activity of antibody-drug conjugate RGX-019-MMAE in MOLM-14 xenograft model. **A**) Experimental design: 0.5 million firefly luciferase GFP+ MOLM-14 cells were injected in NSG mice via tail vein, and leukemia engraftment was measured weekly by bioluminescence imaging (BLI). The mice were treated with vehicle, monoclonal antibody RGX-019 or RGX-019-MMAE at 1 and 5 mg/kg once a week via intraperitoneal injection. **B**) BLI images of the mice treated with indicated doses of RGX-019 or RGX-019-MMAE. **C**) Leukemia progression in mice treated with indicated doses of RGX-019 or RGX-019-MMAE at day 7, 14, 20, 25, and 33. **D**) Dot plot showing leukemia progression in mice treated with the indicated doses of RGX-019 or RGX-019-MMAE on Day 33.** E**) Kaplan-Meier survival plot demonstrates overall survival rates in mice treated with RGX-019 or RGX-019-MMAE (log-rank test). **F**) Bar graph showing weight of the mice treated with the indicated doses of RGX-019 or RGX-019-MMAE on Day 30. *p≤0.05, **p≤0.01, ***p≤0.001, ****p≤0.0001

To validate our findings, we replicated this experiment using MOLM-14 cells expressing firefly luciferase model. 0.5 million cells per mouse (*n* = 5) were injected in NSG mice via tail vein injection. Seven days later, leukemia disease burden was measured using bioluminescence imaging (BLI). Mice were randomized into five treatment groups (Vehicle, 1 mg/kg RGX-019, 5 mg/kg RGX-019, 1 mg/kg RGX-019-MMAE, and 5 mg/kg RGX-019-MMAE, *n* = 5) when the leukemia burden reached 1.5 × 10^9^ photons per second. Imaging and treatments were done once a week. Timeline schematic is shown in Fig. 5A.

Similarly, as seen with the MOLM-13 model, in this MOLM-14 model, we observed that the control antibody, RGX-019, did not inhibit leukemia progression compared to the vehicle-treated group (Fig. 5B). In contrast, we observed that leukemia burden in the mice treated with RGX-019-MMAE was significantly lower, in a dose-dependent manner, compared to those treated with RGX-019, as shown in Fig. 5B-D. In addition, Kaplan–Meier survival analysis showed that both mice treated with 1 mg/kg (*p* = 0.008) and 5 mg/kg (*p* = 0.003) RGX-019-MMAE showed significantly extended survival (Fig. 5E). Notably, no weight loss was observed in mice treated with the ADC, indicating that the antibody conjugate was well tolerated in these mice models (Fig. 5F). These findings further validate the observations we had seen with the Molm-13 GFP model: that treatment with RGX-019-MMAE inhibits leukemia progression in vivo and prolongs the survival of the mice by killing MERTK-expressing AML cells.

### Combination of RGX-019-MMAE and venetoclax enhanced AML cell killing

**Fig. 6:**
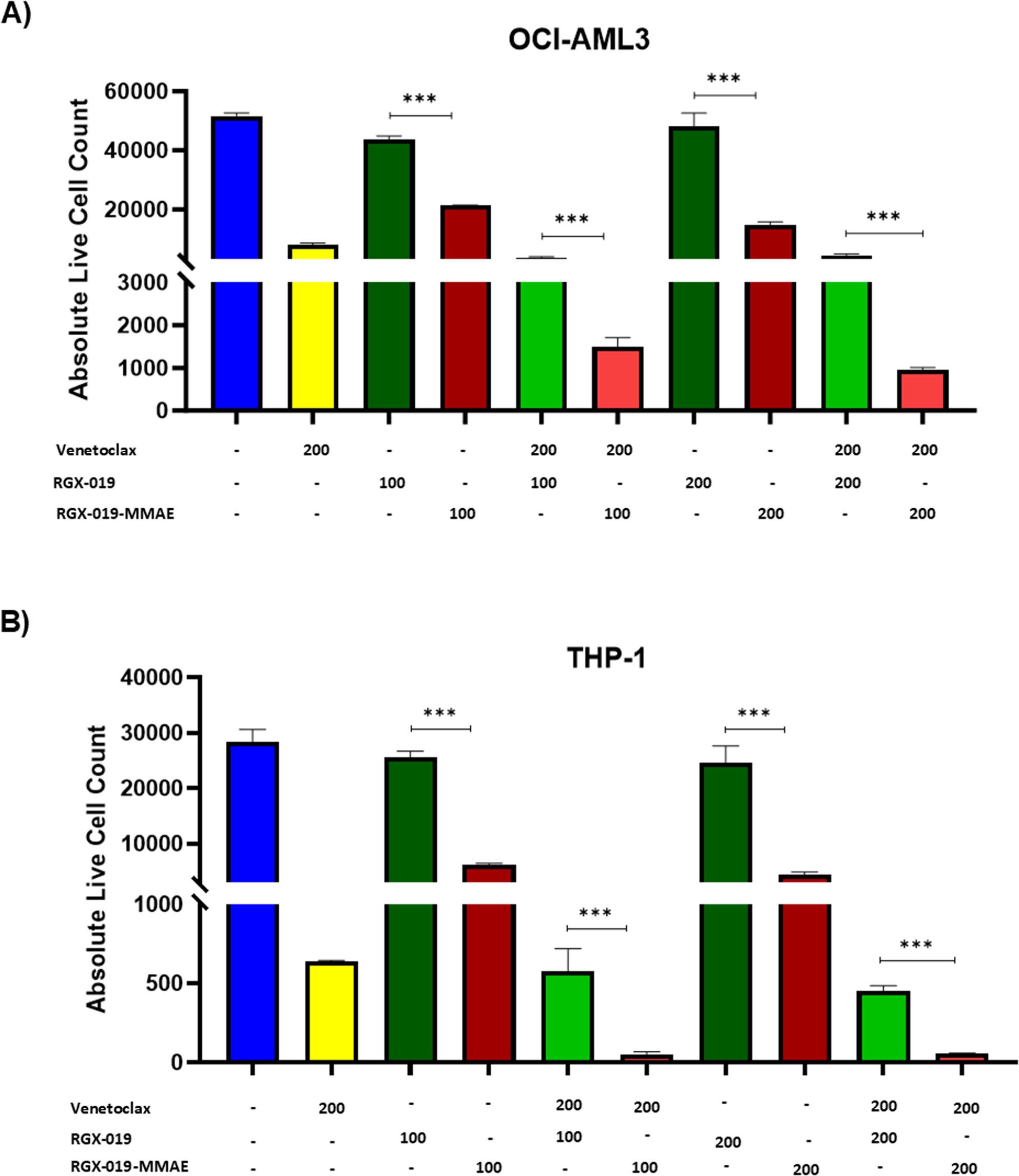
RGX-019-MMAE in combination with venetoclax enhanced AML cell killing. **A**, **B** Bar graphs show the absolute count of live leukemic cells in OCI-AML3 (**A**) and THP-1 (**B**) cell lines treated with indicated concentrations of venetoclax, RGX-019, and/or RGX-019-MMAE. Flow cytometry was used to determine the combinatorial effects of RGX-019-MMAE or monoclonal antibody RGX-019 with venetoclax. Annexin V-FITC was used as an apoptosis marker, and cell viability was determined with DAPI. (Welch one-way ANOVA) *p≤0.05, **p≤0.01, ***p≤0.001, ****p≤0.0001

To determine the synergistic effect of the ADC, we treated MERTK-expressing AML cell lines with RGX-019-MMAE in combination with venetoclax, a BCL2 inhibitor approved for AML treatment. We observed that OCI-AML3 cells treated with RGX-019-MMAE had a significantly lower percentage of live cells than the cells treated with RGX-019 alone (Fig. 6A, S5A). Moreover, combination of venetoclax with RGX-019-MMAE had an additive effect on cell killing compared to cells treated with either agent alone (Fig. 6A, S5A). A similar effect was observed in the THP-1 cell line treated with venetoclax and RGX-019-MMAE (Fig. 6B, S5B). Overall, these findings suggest that RGX-019-MMAE has enhanced AML cell killing when combined with venetoclax.

## Discussion

Here we report that MerTK, a member of the TAM receptor tyrosine kinase family, is overexpressed in AML, particularly in the monocytic subtype. In our preclinical study, the novel MERTK-targeting ADC, RGX-019-MMAE [29] targets AML cells and effectively inhibits their progression in vitro and extended survival in vivo, while no effect was observed on the hematopoietic stem cells. Additionally, we observed a additive effect of combining RGX-019-MMAE with venetoclax, an approved targeted AML therapy. We found that MERTK was overexpressed in monocytic subtypes of AML and expression levels are associated with t(9;11) and inversion 16 cytogenetic abnormalities. These findings are consistent with those of Lee-Sherick et al., who reported that MERTK is overexpressed in 80% of pediatric and 100% of adult AML patients’ blasts at the time of diagnosis [12]. Additionally, several studies have shown that MERTK is overexpressed in AML cell lines, especially monocytic AML cells [12, 31]. Similarly, we observed the highest expression of MERTK in monocytic subtype of Kasumi-1 cell line. A potential reason could be that MERTK plays a significant role in the functional regulation of monocytes and immune cells [32]. Since monocytic subtype of AML arises from monocytic precursors, which have high MERTK expression [33], this could explain why we observed high MERTK expression in Kasumi-1. Therefore, MERTK is an attractive target for monocytic subtypes of AML.

Previous studies showed that knockdown of MERTK in cancer cells led to decreased activity in pro-survival signaling pathways, inhibited colony formation, and induced apoptosis in vitro [13, 14]. In addition, several preclinical studies have described the therapeutic effects of MERTK inhibitors in AML cell lines with or without FLT3-mutations [14, 34, 35]. Here, we demonstrated that RGX-019-MMAE had strong and selective cytotoxicity in AML cell lines and that FLT3-mutated AML cell lines are highly sensitive to RGX-019-MMAE. A possible explanation could be that FLT3-mutated AML cell lines are highly proliferative [36, 37]. The payload of the ADC, MMAE, targets cells that are actively dividing and disrupt the microtubule formation to induce apoptosis. In addition, the GAS6 ligand, which binds to the MERTK receptor, influences FLT3 phosphorylation activity. It has been reported that the depletion of GAS6 results in decreased FLT3 phosphorylation in FLT3-ITD cell lines, which indicates that MERTK may play a critical role in promoting FLT3 status [38, 39]. Moreover, RGX-019-MMAE induces cytotoxicity in a dose dependent manner in primary AML cells ex vivo. Interestingly though, we observed some primary AML cells to be resistant to RGX-019-MMAE. A possible explanation could be the heterogeneity of the cytogenetics and molecular mutations of the patient cohort as well as their previous treatments. Although the IC₅₀ values of RGX-019-MMAE in primary AML samples were relatively high (> 25 nM) compared with other ADCs, such as anti-FLT3 ADCs [40], these finding highlight the need for careful dose optimization in in vivo and clinical studies to maximize therapeutic efficacy while minimizing toxicity.

The development of ADCs offers a promising treatment approach in AML. For example, Gemtuzumab ozogamicin (GO), the first first-generation ADC targeting CD33 on myeloid leukemia cells has shown promising efficacy in newly diagnosed AML [41]. However, there are limitations in safety and efficacy of the effect of this ADC, especially in off-target toxicity affecting normal hematopoietic stem cells. This off-target toxicity can lead to complications such as anemia, bone marrow suppression, risk of infection, etc [42]. Unlike standard therapies, RGX-019-MMAE delivers highly cytotoxic agents selectively to MERTK-expressing leukemia cells while limiting off-target toxicity by sparing normal hematopoietic stem cells because these cells do not express Mer [12, 13, 43]. In our study, RGX-019-MMAE demonstrated a moderate effect of RGX-019-MMAE on immune cells but did not affect PBMC-derived hematopoietic stem cell (HSC) function. Therefore, we anticipate that the remaining/spare HSCs are capable of replenishing the immune cell components affected by the ADC. In addition, the treatment was well tolerated in vivo, as evidenced by the no weight loss in xenograft models.

We also explored the potential effects of RGX-019-MMAE on immune cells given MERTK expression in non-cancerous populations. In vitro studies with primary human NK cells indicated minimal cytotoxicity following ADC treatment, suggesting these cells are largely unaffected. Although T-cell experiments showed high donor-to-donor variability, such variability is commonly observed in primary human T-cell cultures due to differences in donor heterogeneity, cytokine composition, and activation dynamics [44]. MERTK is also expressed on endothelial and retinal pigmented epithelial (RPE) cells where it modulates vascular stability and retinal integrity. Previous in vitro retinal phagocytosis assays and in vivo studies in mouse models expressing a humanized version (extracellular domain) of MERTK showed that RGX-019-MMAE did not abolish phagocytosis by RPE cells nor induce retinal degeneration in these mice [29]. However, safety studies in relevant toxicity models, including cynomolgus monkeys, will be needed to assess potential liabilities, including retinal and endothelial safety, in detail. In vivo studies of ADCs in AML have shown promising results, effectively targeting leukemic cells while minimizing damage to healthy tissues [40, 45, 46]. These studies demonstrate ADCs’ ability to reduce tumor burden, extend survival, and improve treatment efficacy, especially when combined with other therapies [40, 45, 46]. Consistent with these findings, we observed a dose-dependent response to RGX-019-MMAE in both MOLM-13 and MOLM-14 xenograft models, which are aggressive FLT3-mutated models, proving the robustness and high efficacy of the ADC. Although MOLM-13 and MOLM-14 are FLT3-ITD–positive AML cell lines derived from the same patient, they exhibit clear biological differences. MOLM-13 cells display greater dependence on FLT3-ITD signaling, enhanced sensitivity to FLT3 inhibitors, and increased responsiveness to cytokines such as GM-CSF compared to MOLM-14 [47]. Additionally, differences in MERTK expression, growth kinetics, and drug response profiles have been reported and were also observed in our study [48]. Despite their common origin, these cell lines represent biologically distinct AML models. Therefore, we included both xenograft models to validate the reproducibility of RGX-019-MMAE efficacy across related but biologically distinct AML models. In contrast, targeting MERTK with the monoclonal antibody RGX-019 was much less effective, resulting in poor overall survival. This indicates that blocking MERTK signaling by a naked antibody is not sufficient to inhibit leukemia progression, however, adding a toxic payload can significantly enhance anti-tumor efficacy.

The combination of RGX-019-MMAE with a BCL2-inhibitor, venetoclax, showed a significant enhanced effect on AML cell killing in vitro compared to the monoclonal antibody or the ADC alone. OCI-AML3 and THP-1 are resistant cell lines and respond to venetoclax at high concentrations. In addition, some patients are resistant to targeted therapies, including venetoclax [49]. Therefore, we anticipate that patients who are not responding to venetoclax alone could benefit to the combination treatment of RGX-019-MMAE and venetoclax, however, this needs to be explored in future studies. The drug MMAE conjugated with RGX-019 is a microtubule-targeting agent. Disruption of microtubules by MMAE induces mitotic arrest, which in turn triggers apoptotic pathways, including caspase activation and mitochondrial outer membrane permeabilization (MOMP) [50]. However, the efficiency of MOMP and thus apoptosis can be hindered in cells with high levels of the anti-apoptotic protein BCL-2, which acts to preserve mitochondrial integrity and block cell death. To overcome this potential resistance mechanism, we combined RGX-019-MMAE with venetoclax, a selective BCL-2 inhibitor. Venetoclax can sensitize AML cells to apoptosis by neutralizing BCL-2, thereby enhancing the cytotoxic effect initiated by MMAE. Consistent with the previous studies, other microtubule-targeting agents (e.g., vincristine, paclitaxel) have demonstrated modulation of BCL-2 family proteins, including BCL-2, BAX, and BIM [49]. These observations provide a rationale for exploring the combination of RGX-019-MMAE and venetoclax as a potentially therapeutic approach in AML.

There are a fewlimitations to this study. First, the effect of RGX-019-MMAE on leukemia stem cells was not evaluated. Therefore, future studies are needed to assess this population. Second, the higher-than-expected IC₅₀ values of RGX-019-MMAE may be due to low MERTK surface expression in Kasumi-1 and OCI-AML3 cells, which could limit ADC uptake and thereby reduce cytotoxic efficacy. Third, the relatively high IC₅₀ values (> 25 nM) in primary AML samples indicate a narrow therapeutic window, highlighting the need for careful dose optimization in future in vivo and clinical studies. Finally, the efficacy of the RGX-019-MMAE was evaluated only in AML xenograft models, and not in PDX models, due to the limited drug availability; future studies using PDX models will be important to further validate its translational relevance.

## Conclusion

We have investigated the role of targeting AML with an ADC specifically for MERTK, which is overexpressed in a subset of AML. RGX-019-MMAE caused cell death in AML cell lines and primary patient samples. Furthermore, RGX-019-MMAE combined with targeted therapy showed an enhanced anti-leukemic effect. Finally, RGX-019-MMAE significantly increased survival duration in vivo. Our data indicates that MERTK is a clinically promising target for ADC therapies, especially in AML patients with monocytic leukemia. 

## Supplementary Information

Supplementary Material 1: Table S1. AML patient characteristics used for RPPA analysis.

Supplementary Material 2: Table S2. Molecular mutations and translocations in AML patients used for RPPA analysis.

Supplementary Material 3: Fig S1: MERTK expression in total AML cell population and normal bone marrow derived CD34⁺ cells. MERTK levels were measured by RPPA in whole-cell lysates from AML patient samples and normal bone marrow-derived CD34⁺ cells. Expression values are shown as log₂-fold change relative to normal CD34⁺ cells. Cells were stained with anti-MERTK monoclonal antibody (ab52968).

Supplementary Material 4: Fig S2: Efficacy of MMAE in AML cell lines. A-B) Bar graph showing the percentage of relative luminescence in Kasumi 1 (A) and OCI-AML3 (B) cells treated with the indicated concentrations of free MMAE. Data are plotted as mean values with error bars representing standard error (Student unpaired t-test) *p≤0.05, **p≤0.01, ***p≤0.001, ****p≤0.0001.

Supplementary Material 5Fig. S3: RGX-019-MMAE internalization in AML cell lines. A) Bar graph showing the pHrodo MFI scores of NOMO-1 and Kasumi-1 AML cells treated Fc receptor block and RGX-019-MMAE labeled with pHrodo.

Supplementary Material 6: Fig. S4: The antibody-drug conjugate, RGX-019-MMAE, induced cytotoxic activity in AML primary cells. A) Overlay plot showing MERTK expression in primary cells. Cells were stained with anti-MERTK-APC antibody (blue) or were unstained (red), and MERTK expression was measured by flow cytometry. B-D) Bar graphs show the percentage of relative luminescence in primary AML cells RGX356 (B), RGX574 (C), RGX228 (D), and NK cells treated with the indicated concentrations of RGX-019-MMAE and monoclonal antibody RGX-019. Data are plotted as mean values with error bars representing standard error (Student unpaired *t*-test) *p≤0.05, **p≤0.01, ***p≤0.001, ****p≤0.0001

Supplementary Material 7: Fig. S5: The antibody-drug conjugate RGX-019-MMAE in combination with venetoclax enhanced AML cells killing. Dot plot showing percentage of apoptotic cells in OCI-AML3 (A) and THP-1 (B) cell lines treated with indicated concentration of venetoclax, monoclonal antibody RGX-019 and/or RGX-019-MMAE. Flow cytometry was used to determine the effects of RGX-019-MMAE or RGX-019 in combination with venetoclax (Welch one-way ANOVA) *p≤0.05, **p≤0.01, ***p≤0.001, ****p≤0.0001

## Data Availability

All of the data supporting the findings of this study are available from the corresponding author on reasonable request.

## Abbreviations

AML: Acute Myeloid Leukemia
ADCs: Antibody-Drug Conjugates
MERTK: MER proto-oncogene tyrosine kinase
TAM: Tyro3, Axl, MERTK
GAS6: Growth Arrest Specific 6
RPPA: Reverse-phase protein array
NK: Natural Killer
CI: Combination Index
eGFP-FFluc: Enhanced Green Fluorescent Protein Firefly Luciferase
BLI: Bioluminescence Imaging
WBC: White Blood Count
MMAE: Monomethyl Auristatin E
GO: Gemtuzumab Ozogamicin
