# Supplementary figures and images for: RGX-019-MMAE inhibits leukemia progression by targeting MER proto-oncogene tyrosine kinase (MERTK) in acute myeloid leukemia
Source: J Exp Clin Cancer Res. 2026 Mar 28;45:81. doi: 10.1186/s13046-026-03657-y (PMC13032267; doi:10.1186/s13046-026-03657-y)

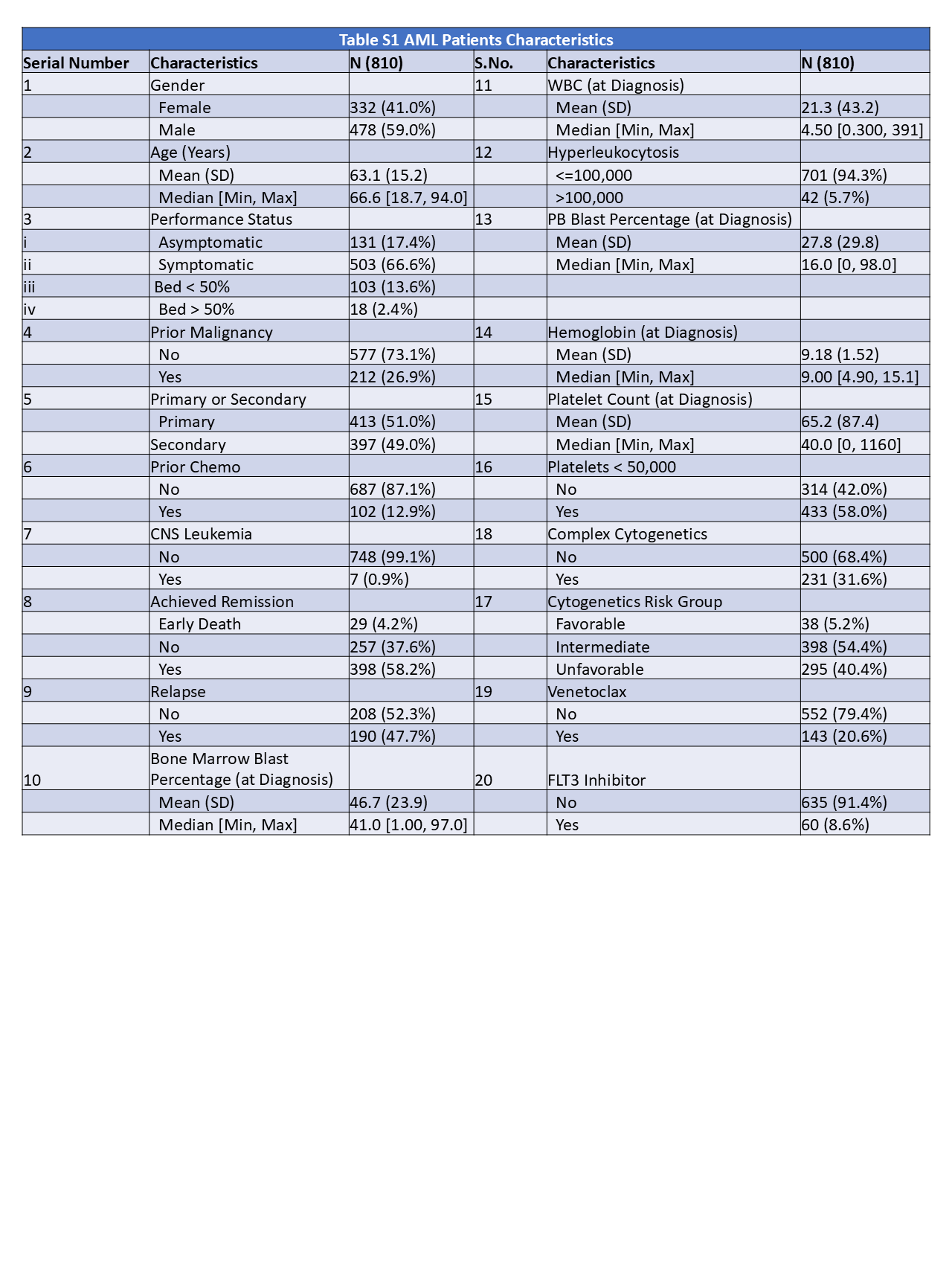

Supplement: Supplementary file 1 — Supplementary Material 1: Table S1. AML patient characteristics used for RPPA analysis. [file 13046_2026_3657_MOESM1_ESM.tif]

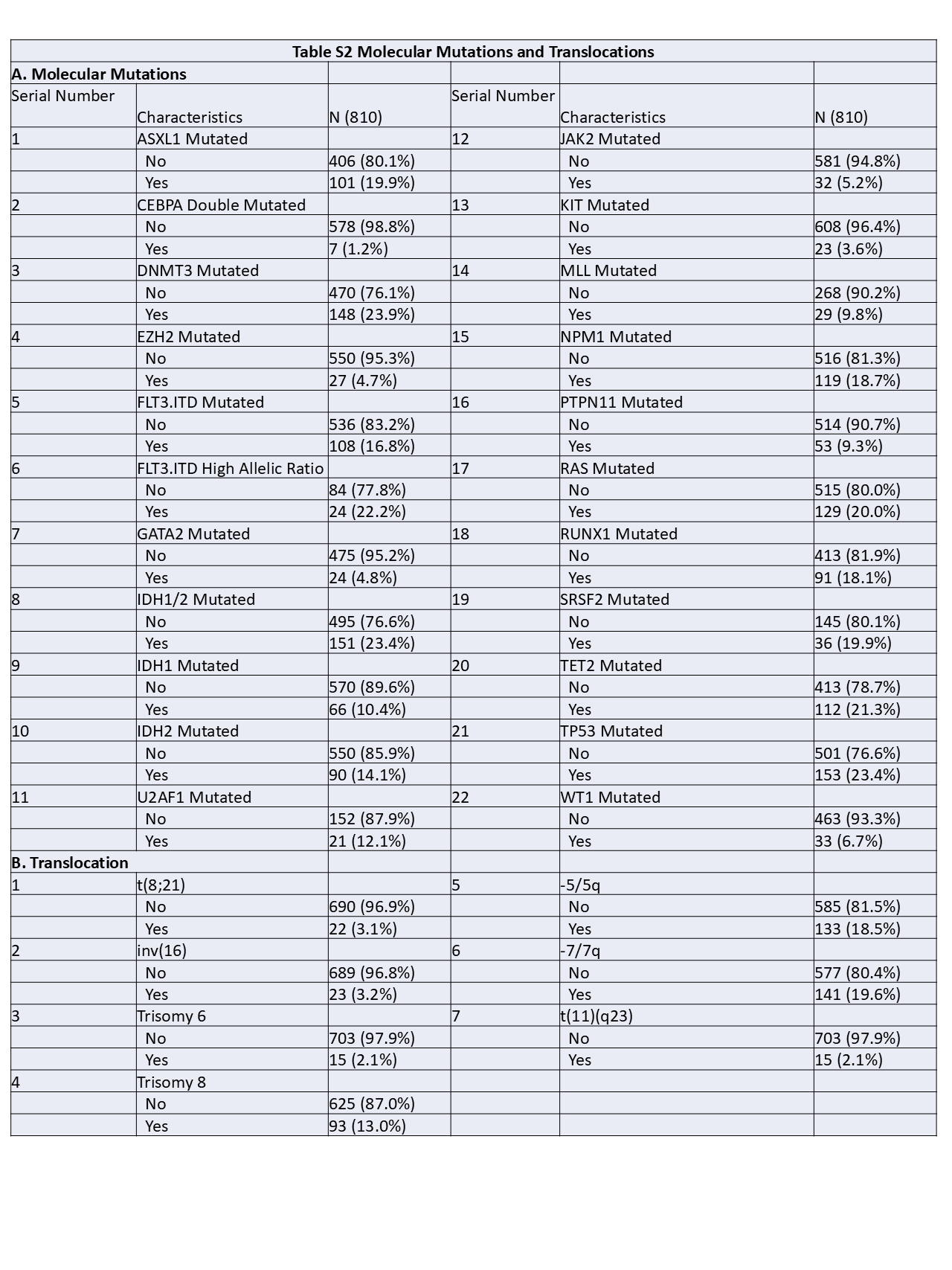

Supplement: Supplementary file 2 — Supplementary Material 2: Table S2. Molecular mutations and translocations in AML patients used for RPPA analysis. [file 13046_2026_3657_MOESM2_ESM.tif]

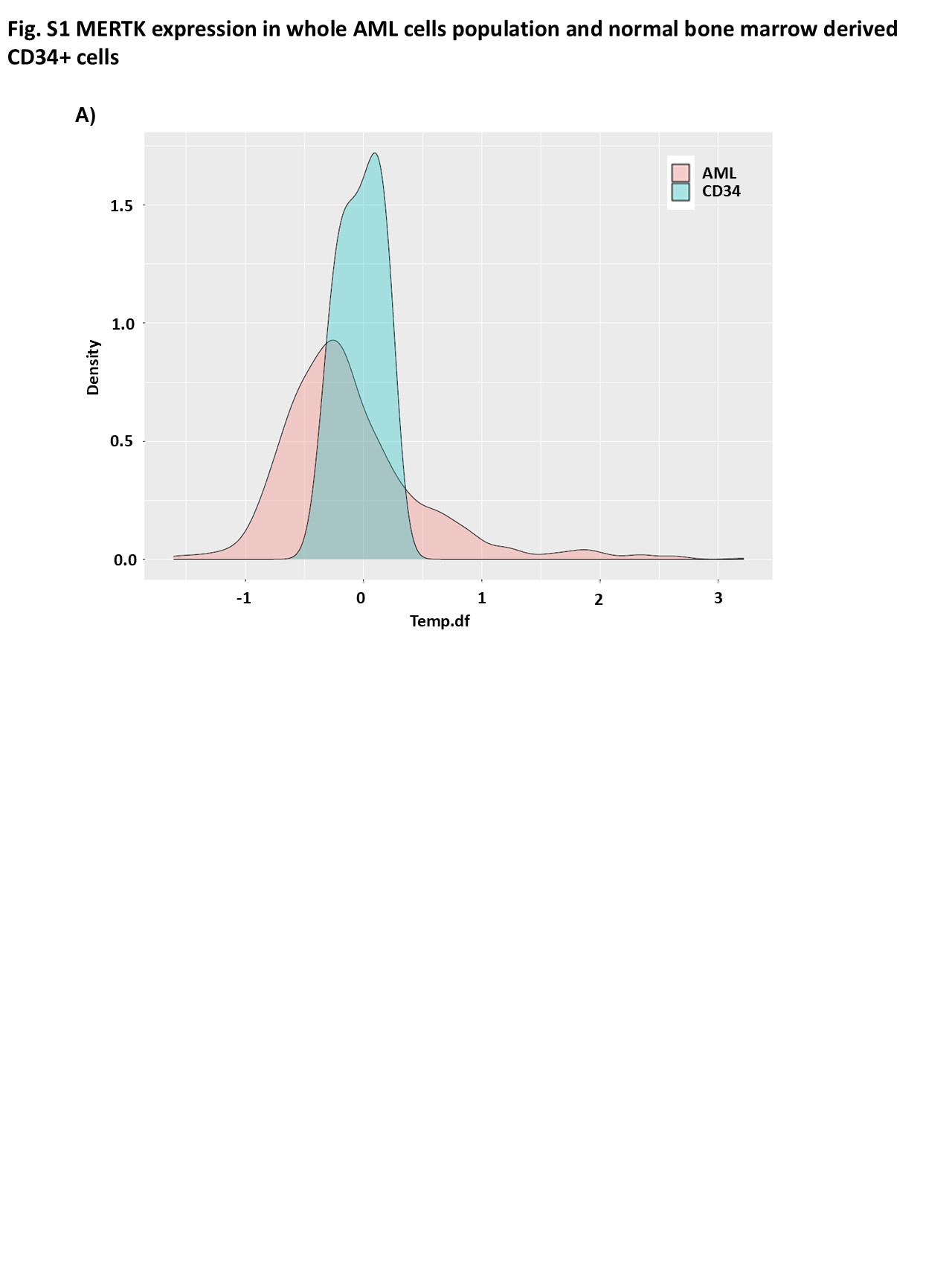

Supplement: Supplementary file 3 — Supplementary Material 3: Fig S1: MERTK expression in total AML cell population and normal bone marrow derived CD34⁺ cells. MERTK levels were measured by RPPA in whole-cell lysates from AML patient samples and normal bone marrow-derived CD34⁺ cells. Expression values are shown as log₂-fold change relative to normal CD34⁺ cells. Cells were stained with anti-MERTK monoclonal antibody (ab52968). [file 13046_2026_3657_MOESM3_ESM.tif]

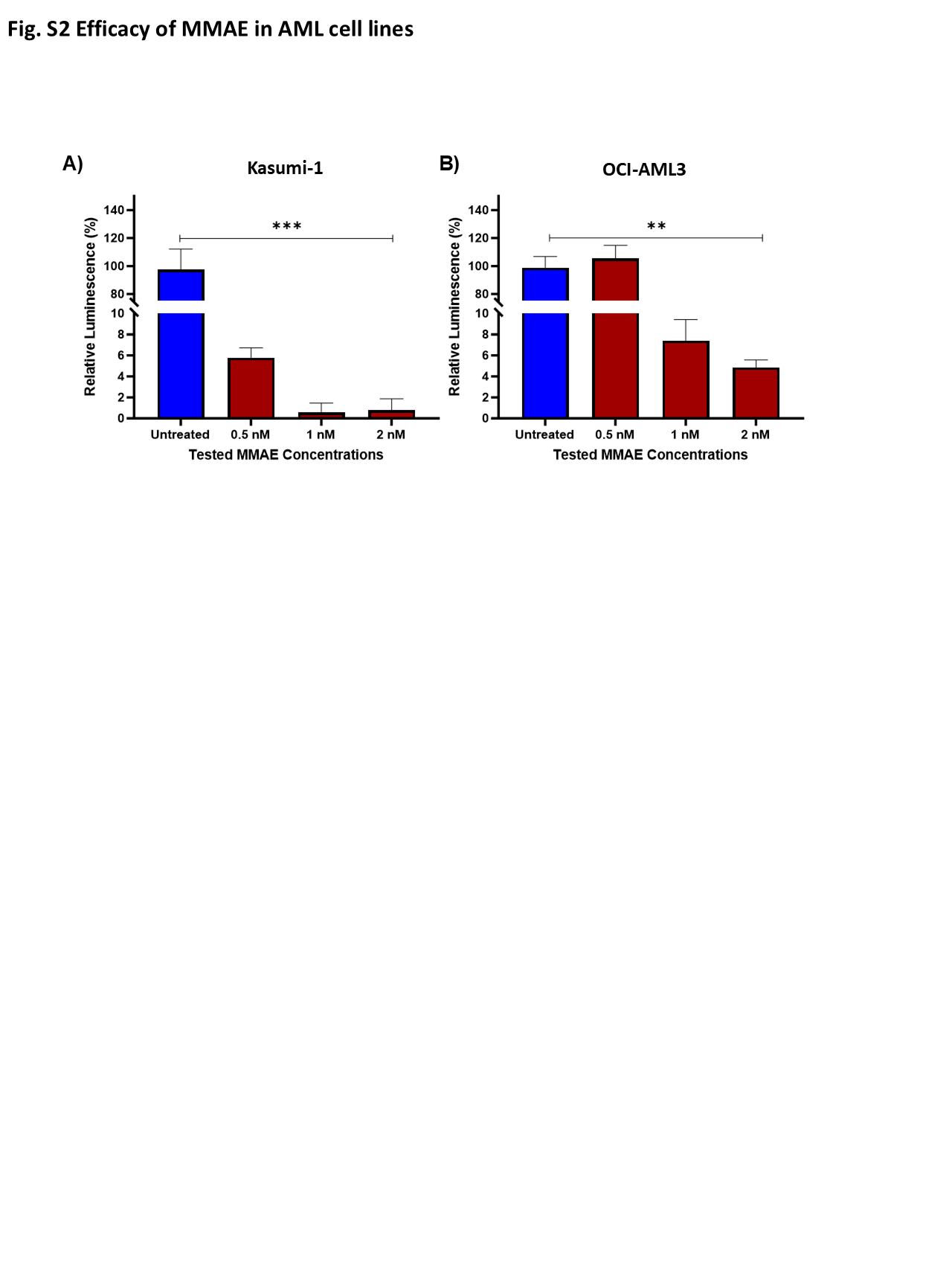

Supplement: Supplementary file 4 — Supplementary Material 4: Fig S2: Efficacy of MMAE in AML cell lines. A-B) Bar graph showing the percentage of relative luminescence in Kasumi 1 (A) and OCI-AML3 (B) cells treated with the indicated concentrations of free MMAE. Data are plotted as mean values with error bars representing standard error (Student unpaired t-test) *p≤0.05, **p≤0.01, ***p≤0.001, ****p≤0.0001. [file 13046_2026_3657_MOESM4_ESM.tif]

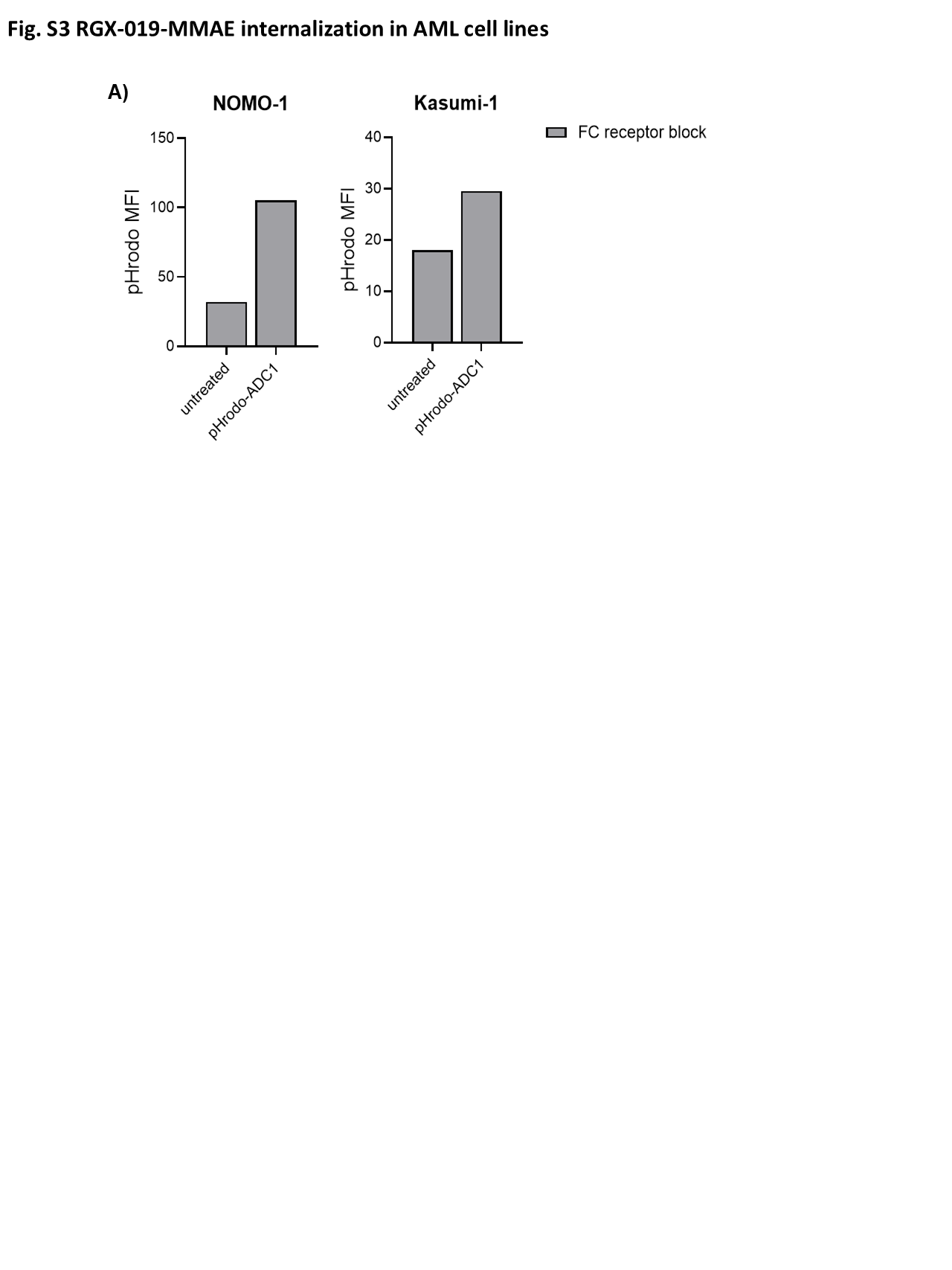

Supplement: Supplementary file 5 — Supplementary Material 5Fig. S3: RGX-019-MMAE internalization in AML cell lines. A) Bar graph showing the pHrodo MFI scores of NOMO-1 and Kasumi-1 AML cells treated Fc receptor block and RGX-019-MMAE labeled with pHrodo. [file 13046_2026_3657_MOESM5_ESM.tif]

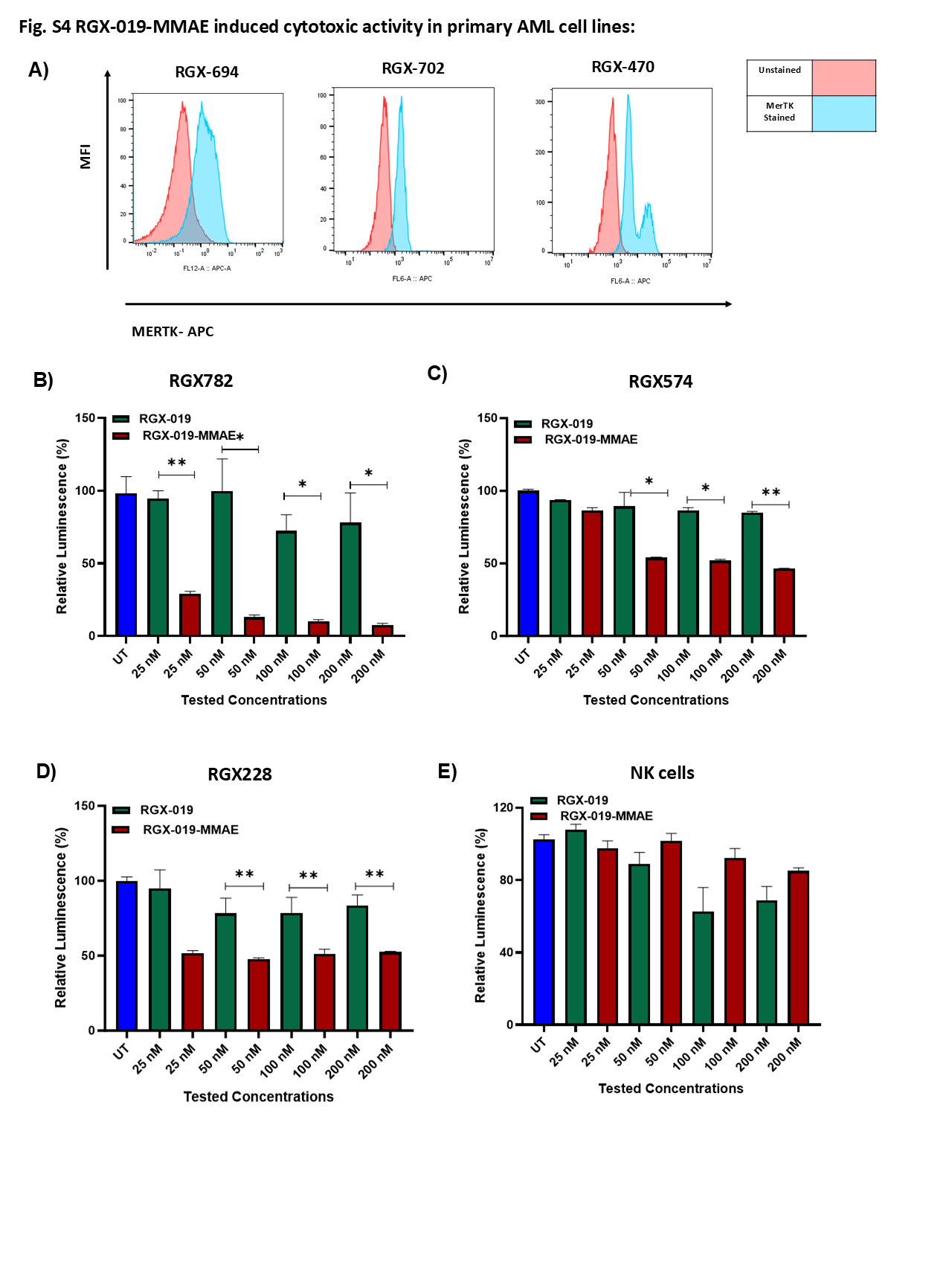

Supplement: Supplementary file 6 — Supplementary Material 6: Fig. S4: The antibody-drug conjugate, RGX-019-MMAE, induced cytotoxic activity in AML primary cells. A) Overlay plot showing MERTK expression in primary cells. Cells were stained with anti-MERTK-APC antibody (blue) or were unstained (red), and MERTK expression was measured by flow cytometry. B-D) Bar graphs show the percentage of relative luminescence in primary AML cells RGX356 (B), RGX574 (C), RGX228 (D), and NK cells treated with the indicated concentrations of RGX-019-MMAE and monoclonal antibody RGX-019. Data are plotted as mean values with error bars representing standard error (Student unpaired t-test) *p≤0.05, **p≤0.01, ***p≤0.001, ****p≤0.0001 [file 13046_2026_3657_MOESM6_ESM.tif]

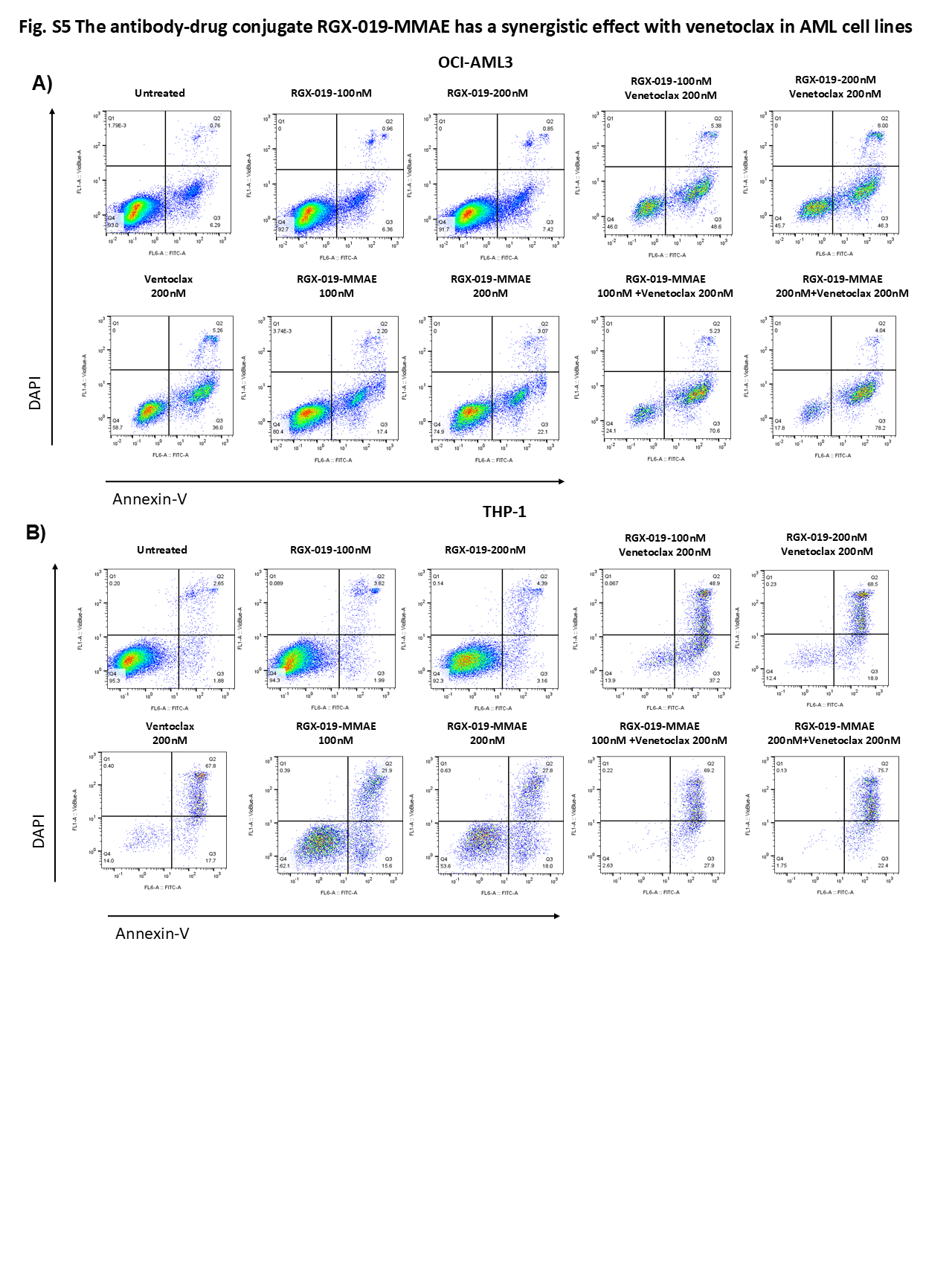

Supplement: Supplementary file 7 — Supplementary Material 7: Fig. S5: The antibody-drug conjugate RGX-019-MMAE in combination with venetoclax enhanced AML cells killing. Dot plot showing percentage of apoptotic cells in OCI-AML3 (A) and THP-1 (B) cell lines treated with indicated concentration of venetoclax, monoclonal antibody RGX-019 and/or RGX-019-MMAE. Flow cytometry was used to determine the effects of RGX-019-MMAE or RGX-019 in combination with venetoclax (Welch one-way ANOVA) *p≤0.05, **p≤0.01, ***p≤0.001, ****p≤0.0001 [file 13046_2026_3657_MOESM7_ESM.tif]
